# PD08 Applying An Adaptive Approach To Address The Challenge Of Health Technology Assessment Appraisal Diversity On Medical Devices

**DOI:** 10.1017/S0266462325101463

**Published:** 2025-12-29

**Authors:** Rachele Busca, Jana Boleckova

## Abstract

**Introduction:**

Health technology assessment (HTA) of Class III implantable medical devices (MDs) in Europe is conducted at the national or regional level. Despite common clinical evidence, sometimes appraisal results and recommendations vary significantly, affecting patient access to innovative technologies. An adaptive approach to HTA for MDs will need to be consistent with current expert thinking on adaptive approaches in HTA methodology.

**Methods:**

The websites of HTA agencies across 13 European countries were searched for HTA reports published between 1 January and 31 December 2023 on high-risk cardiovascular MDs used to treat structural heart disease. The goal was to identify the classes of technologies assessed by different HTA agencies and compare the submitted evidence and decisions. Since assessments are sometimes repeated across different years, we reviewed all available submissions for the selected innovations from the CE mark up to 2023 to follow the evolution of appraisals and decisions on the same technology.

**Results:**

Eighty reports from 11 countries were identified, with 23 on structural heart disease from Austria, France, Italy, and Spain. The main diseases involved heart valves, septal defects, and heart failure. In 2023, two agencies reassessed technologies for mitral valve regurgitation (MVR). Austria’s HTA gave negative recommendations to the first device CE marked in 2008 in fourth consecutive assessments, despite three randomized controlled trials (RCTs). A fifth reassessment is planned for 2026. France has conducted 26 assessments since 2015 on MVR devices (due to design changes, indication expansions, generation changes, economic assessments, new devices entering the market, and life cycle reassessment), with a positive recommendation in 2015, which was supported by two single-arm studies and a registry. A second device, CE marked in 2019, was recommended by France in 2023 based on a single-arm trial, an RCT, and two registries.

**Conclusions:**

The number of HTAs performed on MDs remains low, particularly for high-risk device classes. There was no consistency in the number of HTAs performed between countries, nor in the evidence required for a successful assessment. These differences lead to unequal access to specialized treatment for patients in different countries and highlight the need for a unified European HTA approach.

